# Right Heart Size and Right Ventricular Reserve in Pulmonary Hypertension: Impact on Management and Prognosis

**DOI:** 10.3390/diagnostics10121110

**Published:** 2020-12-21

**Authors:** Ekkehard Grünig, Christina A. Eichstaedt, Rebekka Seeger, Nicola Benjamin

**Affiliations:** 1 Centre for Pulmonary Hypertension, Thoraxklinik Heidelberg gGmbH at Heidelberg University Hospital, Röntgenstrasse 1, 69126 Heidelberg, Germany; christina.eichstaedt@med.uni-heidelberg.de (C.A.E.); Rebekka.Seeger@med.uni-heidelberg.de (R.S.); Nicola.Benjamin@med.uni-heidelberg.de (N.B.); 2 Translational Lung Research Centre Heidelberg (TLRC), German Centre for Lung Research (DZL), 69126 Heidelberg, Germany; 3 Institute of Human Genetics, Heidelberg University, Im Neuenheimer Feld 366, 69120 Heidelberg, Germany

**Keywords:** pulmonary arterial hypertension, right heart size, right ventricular reserve, prognosis

## Abstract

Various parameters reflecting right heart size, right ventricular function and capacitance have been shown to be prognostically important in patients with pulmonary hypertension (PH). In the advanced disease, patients suffer from right heart failure, which is a main reason for an impaired prognosis. Right heart size has shown to be associated with right ventricular function and reserve and is correlated with prognosis in patients with PH. Right ventricular reserve, defined as the ability of the ventricle to adjust to exercise or pharmacologic stress, is expressed by various parameters, which may be determined invasively by right heart catheterization or by stress-Doppler-echocardiography as a noninvasive approach. As the term “right ventricular contractile reserve” may be misleading, “right ventricular output reserve” seems desirable as a preferred term of increase in cardiac output during exercise. Both right heart size and right ventricular reserve have been shown to be of prognostic importance and may therefore be useful for risk assessment in patients with pulmonary hypertension. In this article we aim to display different aspects of right heart size and right ventricular reserve and their prognostic role in PH.

## 1. Introduction

Pulmonary arterial hypertension (PAH) is a chronic disease, which is characterized by progressive remodeling of the pulmonary arteries, leading to increased pulmonary arterial pressure and resistance. In the advanced disease, patients suffer from right heart failure, which is the reason for an impaired prognosis [1,2].

Various parameters reflecting right heart size and right ventricular function have been shown to be prognostically important in patients with PAH [3]. Ventricular reserve is defined as the ability of the ventricle to adjust to exercise or pharmacologic stress. In this regard, contractile reserve refers to changes in systolic function [3]. Determining factors of contractile reserve include ventricular contractility, elasticity, remodeling, afterload and perfusion [3].

Furthermore, PAH-targeted treatment with riociguat has shown a beneficial effect on right heart size, while exercise training improved right ventricular (RV) reserve.

To cover different aspects of RV function and right heart size, various definitions have been described.

In this article we aim to display different aspects of RV size and reserve and their prognostic role in PAH.

## 2. Materials and Methods

### 2.1. Literature Search

Literature was searched in PubMed and the Cochrane Library including randomized controlled trials, clinical trials, meta-analyses, systematic reviews and reviews. Articles that were not written in English or German were excluded. Keywords for literature search in October 2020 were “pulmonary hypertension” in combination with “right ventricular reserve”, “contractile reserve”, “right ventricular size”, “right heart size” with and without “prognosis”. Reference lists of included studies were searched for further suitable publications.

### 2.2. Study Selection

Literature was reviewed for content. Publications which did not include patients with PAH and assessment of right heart size or RV reserve were excluded.

## 3. Results

### 3.1. Definitions of Right Heart Size

**Figure 1:**
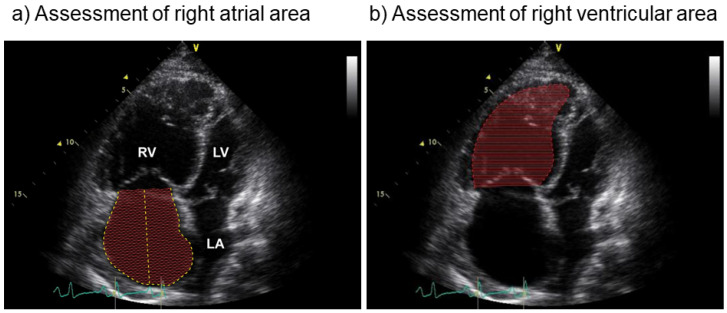
RA (right atrial) and RV (right ventricular) areas are assessed by transthoracic echocardiography in apical 4-chamber view (The 4-chamber view also displays LV (left ventricle) and LA (left atrium). For the assessment, the margins of the right atrium and the right ventricle are traced to calculate their area. In this figure, RA (**a**) and RV (**b**) areas are shown filled out with lines.

The adaptation of the right ventricle to cope with increased resistance of the pulmonary vasculature has been shown to be an important predictor of survival [2,4]. In advanced PAH increased pulmonary load leads to RV dilatation in order to maintain stroke volume (heterometric adaptation) [5]. RV dilatation may be assessed by measurements of right heart size using echocardiography (right atrial (RA) and RV area, Figure 1) or magnetic resonance imaging (RA and RV volume). These parameters may indicate pulmonary load and are therefore also used as monitoring parameters for follow-up assessments in patients with pulmonary hypertension [6].

Right heart size may furthermore be estimated by RA and RV volumes using 3D echocardiography. Both end-diastolic and end-systolic volumes may be assessed with reference ranges of end-diastolic volume 87 mL/m^2^ for males and 74 mL/m^2^ for females and end systolic volume 45 mL/m^2^ for males and 36 mL/m^2^ for females [7]. RA and RV area are determined by age, sex, body surface area and athletic status (conduct of endurance training) [8].

The interaction between RV load and pump function has shown to be a crucial factor to determine RV-arterial coupling [5,9,10]. In this regard, the elasticity of the pulmonary arteries is a determining factor of the distensibility of the pulmonary vascular bed and is expressed as pulmonary arterial compliance (or capacitance). RV function adaptation to afterload is initially systolic. Increased right heart dimensions in PH-patients reflects water overload most often due to reduced RV contractility to match the increased afterload, first during exercise [11,12], then at rest. For the correct interpretation of increased RV and RA areas/dimensions several parameters should therefore be analyzed: systolic RV function (tricuspid annular plane systolic excursion (TAPSE), etc.) and afterload (pulmonary vascular resistance, slope of pulmonary artery pressure—flow relationships), and their integration in the assessment of RV-pulmonary arterial coupling (TAPSE/systolic pulmonary arterial pressure, TAPSE/end-systolic volume or area, fractional area change/end-systolic area, etc.) [13]. Increase in RV size was found to be associated with a decrease of >50% end-systolic to arterial elastance [14], resembling advanced stages of the disease with an ejection fraction of <35–40% [15]. However, in screening assessments of patients at risk for PH very mildly enlarged RA- size and/or RV-size (area > 16 cm^2^) occurred in very early disease and can therefore be used as an early marker of RV dysfunction [16,17,18].

In patients with PAH, right heart size and function showed a significant correlation, with impaired RV reserve, measured by cardiac index increase during exercise, if they presented with enlarged RA and/or RV area (above a median of 16 [8] and 20 cm^2^ [19], respectively). In this study, patients with enlarged RV area presented with significantly lower RV stroke volume and RV area was identified as the only independent predictor of RV output reserve [20].

### 3.2. Definitions of Right Ventricular Reserve

**Figure 2:**
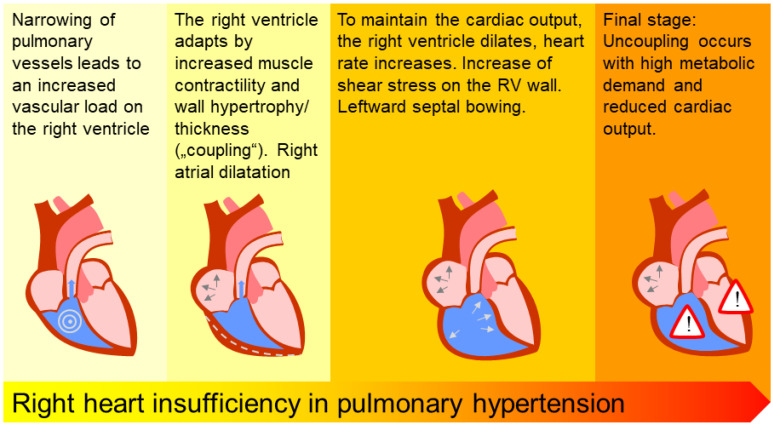
Pathophysiology of right heart insufficiency, adapted from [5].

The ability of the ventricle to adjust to exercise or pharmacologic stress becomes impaired by the pathophysiologic changes of the pulmonary vessels, the right atrium and the right ventricle in pulmonary hypertension (Figure 2).

Several parameters have been introduced to estimate RV reserve and to cover different aspects of its pathophysiological determinants (Table 1).

Pulmonary vascular or cardiovascular reserve may be expressed as mean pulmonary arterial pressure increase relative to cardiac output increase, indicating the pressure-flow relationship during exercise. An increase >3 mmHg/L/min is defined as abnormal [21]. Patients with manifest PAH have shown an impaired pulmonary vascular reserve (with higher pressure increase of mPAP relative to cardiac output) compared to healthy controls (*p* < 0.05) [22]. Asymptomatic family members of PAH patients carrying a bone morphogenetic protein receptor type 2 (BMPR2) mutation showed significantly more often a hypertensive response of systolic pulmonary arterial pressure during low exercise compared to controls [23]. Activating BMPR2 mutations have been shown to be associated with PAH and lead to increased cell growth and reduced apoptosis.

RV pump function during exercise, expressed as RV output reserve, displays the capacity of the RV to adjust its systolic function to exercise and pulmonary loading [9]. The ability to increase cardiac output during exercise has often been defined as right ventricular contractile reserve. These two terms may be used as synonyms. “Contractile” reserve may be misleading, though, as it might also refer to the contractility of the right ventricular muscles. Consequently, in this regard the term “right ventricular output reserve” seems preferable, as it clearly refers to an increase in cardiac output. In this regard RV output reserve is defined as increase of cardiac index during exercise measured by right heart catheterization.

RV free wall longitudinal strain, which is an indicator of RV function at rest, is calculated as the mean of the RV lateral basal, mid, and apical segments, with exclusion of the septal segments. In one study, RV contractile reserve was assessed as the difference in RV free wall strain at rest and during a leg-positive pressure stress [24]. In this study of 43 pulmonary hypertension patients, RV contractile reserve was significantly lower in patients with changes in left ventricular stroke volume < 3.3 mL.

Pulmonary arterial elasticity plays an important role for right ventricular function and maintains low pulse pressure and low pulsatile afterload for the right ventricle. Pulmonary arterial compliance (PAC) decreases in PAH [25] and correlates with pulmonary hypertension severity [26]. The calculation of PAC by Jain et al. has presented as a simple and practical method for estimating pulmonary arterial elasticity [25]. PAC is crucial for passive arterial expansion, enabling accommodation of much of the RV stroke volume. It also helps maintaining diastolic pulmonary blood flow due to arterial recoil. A decrease in PAC increases RV pulsatile afterload [2,27], leading to an impaired RV function and right heart failure [28,29]. Decreased PAC has been shown to be a determining factor of RV failure, dilatation and hypertrophy [30] and independent from improvements of pulmonary vascular resistance during targeted PAH treatment [2,28,31]. Rest-to-exercise response in end-systolic elastance is a further invasive assessment to investigate exertional contractile reserve in pulmonary hypertension [11].

Noninvasive approaches to estimate RV output reserve include change of systolic pulmonary arterial pressure during exercise [32] and a combination of echocardiographic parameters including change in tricuspid annular plane systolic excursion (ΔTAPSE), change in RV fractional area change (ΔRVFAC), and change in Doppler-derived tricuspid lateral annular peak systolic velocity (ΔS’) [33,34]. The idea behind this concept is, that patients with low cardiac output increase during exercise cannot increase their systolic pulmonary arterial pressure. In the first study using stress-Doppler-echocardiography the group of patients who revealed a low exercise-induced systolic pulmonary arterial pressure increase, i.e., a response below the median value of 30 mmHg, showed a significantly lower mean 6-min walking distance, mean peak VO_2_/kg, lower mean heart rate, N-terminal pro brain natriuretic peptide, RA area and a lower 1-, 3- and 4-year survival rate. [32] The study revealed that systolic pulmonary arterial pressure increase during low workload exercise assessed by echocardiography was an independent prognostic factor. We concluded that exercise-induced systolic pulmonary arterial pressure increase could be an estimate of right ventricular contractile (output) reserve.

In further echocardiographic studies [33,34] these results were confirmed. Compared with controls, pulmonary hypertension and PAH patients presented with a significantly lower combined change in tricuspid annular plane systolic excursion (ΔTAPSE), change in RV fractional area change (ΔRVFAC), and change in Doppler-derived tricuspid lateral annular peak systolic velocity (ΔS’)) [33,34]. RV function and RV output reserve showed to be associated with maximal exercise capacity [33].

In patients with systemic sclerosis and manifest PAH, RV output reserve measured by right heart catheterization at rest and during exercise has shown to be depressed compared to idiopathic PAH and was associated with ventricular-pulmonary uncoupling and dilation of RV area [12]. Furthermore, patients with only mildly elevated pulmonary arterial pressures (mean pulmonary arterial pressure 21–24 mmHg) already showed significantly impaired RV output reserve and significantly lower pulmonary arterial compliance than patients with normal mean pulmonary arterial pressures [18].

### 3.3. Prognostic Importance of Right Heart Size

Due to its prognostic value, RA area is included in the parameters for risk stratification of patients with PAH in the current guidelines [6,35]. While RA area < 18 cm^2^ has been shown to be associated with a good prognosis, areas between 18–25 cm^2^ bear a moderate and >25 cm^2^ a high mortality rate [6,35].

Right heart size, assessed as RA [8,36,37] and RV area or volumes, have repeatedly been proven of prognostic significance in pulmonary hypertension [2,38], whereas their impact on RV contractility needs more data. During the last years, magnetic resonance imaging studies have shown increased RV volumes (assessed at the endsystole or diastole) were significant predictors of outcome and were also associated with an impaired RV stroke volume [39,40,41]. Furthermore, increasing RV volumes during follow-up do often occur with further clinical signs of disease progression, leading to death or transplantation, whereas stable RV volumes indicate a stable clinical course [40]. In this study, during the same time, hemodynamic parameters remain unchanged and may therefore not as well indicate disease progression, while RV ejection fraction showed a decline in patients with further enlargement of RV volumes [40].

An analysis of the French PAH registry confirmed these findings by showing that stroke volume index and RA pressure present as independent predictors of death or lung transplantation after PAH treatment initiation [42].

### 3.4. Prognostic Importance of Right Ventricular Reserve

RV output reserve has demonstrated prognostic importance in patients with PH [43,44]. A non-invasive assessment of right ventricular reserve is the ability of the RV to increase pulmonary arterial pressure during exercise, as assessed by systolic pulmonary arterial pressure increase. Systolic pulmonary arterial pressure increase during exercise has presented as independent predictor of survival and showed best predictive power in combination with peak oxygen consumption [32].

In patients with PH, systolic pulmonary arterial pressure increase < 30 mmHg was associated with worse survival compared to pressure increase > 30 mmHg [32]. A lower systolic pulmonary arterial pressure increase may therefore describe an impaired ability of the right ventricle do adapt to pulmonary load and exercise and to further increase pressure and pulmonary blood flow.

In comparison with pulmonary vascular resistance, being the most common hemodynamic parameter to characterize resistance of the pulmonary vascular bed, pulmonary arterial compliance (PAC) has shown superiority in the prediction of mortality in patients with PAH [45]. PAC was also associated with a higher hazard ratio than mPAP and cardiac index [45]. This result was also confirmed in patients with PH due to heart failure with preserved ejection fraction [46] and in patients with PH due to left heart failure (World Health Organization Group II) [46,47,48,49]. Change of PAC during treatment has shown to be a superior predictor of outcome than PAC at baseline with a hazard ratio of 4.21 (CI 1.77–10.02, *p* = 0.004) in the multivariate analysis [50].

In this regard, also increased pulmonary artery stiffness, assessed by cardiac magnetic resonance imaging, was associated with impaired survival in PAH patients [51].

### 3.5. Treatment Effects on Right Heart Size and Function

Right heart size can be reduced by preload control, i.e., diuretics, and targeted treatment as previously described by Vizza et al., which can markedly improve outcome [52]. In a retrospective analysis of Riociguat studies including 39 patients with PAH and CTEPH, treatment with Riociguat significantly improved right heart size with significant decrease of mean RV area after 3 (−2.1 ± 3.9 cm^2^, equals −7.4 ± 15.3%, *p* = 0.002), 6 (−4.2 ± 3.2 cm^2^, equals −16.1 ± 11.5%, *p* < 0.001) and 12 months (−5.9 ± 4.6 cm^2^, equals −22.1 ± 14.2%, *p* < 0.001) compared to baseline. RA area significantly decreased after 12 months (−3.5 ± 4.1 cm^2^, equals −16.8 ± 19.2%, *p* < 0.001) and TAPSE significantly improved after 6 (+2 ± 4.7, equals 12 ± 25.8%, *p* = 0.025) and 12 months (+3.6 ± 5.4, equals 21.0 ± 29.6%, *p* = 0.002) [53]. These results were confirmed in a larger-scaled study with analysis of 71 PAH and CTEPH patients with significant reduction of RA and RV area after 6 months [54]. After 12 months, patients receiving riociguat therapy showed a significant reduction in RA (−2.6 ± 4.4 cm^2^, 95% CI −3.84, −1.33; *p* < 0.001, *n* = 49) and RV area (−3.5 ± 5.2 cm^2^, 95% CI −5.1, −1.9; *p* < 0.001; *n* = 44) [54].

Though reverse remodeling of the right heart is associated with functional improvement, moderate improvements in RV function may be lacking decrease of right heart size [55]. RV end-diastolic area has presented as a useful prognostic parameter, which may even provide an estimate of morbidity and mortality irrespective of baseline risk assessment [55].

Parenteral prostacyclin has shown to slightly improve PAC, while improving 6-minute walking distance [29]. Improvement of RV volumes has been shown in two PAH-treatment studies, suggesting an improvement in RV pump function [41,56].

In addition, a post-hoc analysis of right heart catheterization data showed a significant improvement of PAC by exercise training in PAH and CTEPH [57]. Low-dose exercise training at 4–7 days/week significantly improved PAC (training group 0.33 ± 0.65 mL/mmHg vs. control group −0.06 ± 1.10 mL/mmHg; mean difference 0.39 mL/mmHg, 95% confidence interval (CI) 0.15 to 0.94 mL/mmHg; *p* = 0.004) and stroke volume (training group 9.9 ± 13.4 mL/min vs. control group −4.2 ± 11.0 mL/min; mean difference 14.2 mL, 95% CI 6.5 to 21.8 mL; *p* < 0.001) in the training vs. control group [57]. These findings suggest that supervised exercise training might improve RV function and PAC at the same time.

## 4. Conclusions

Right heart size has shown to be associated with right ventricular function and reserve and is associated with an impaired survival in patients with PAH. Right ventricular reserve is expressed by various parameters, which may be determined invasively by right heart catheterization or by stress-Doppler-echocardiography as a noninvasive approach. A combination of these parameters offers to cover different aspects of right ventricular reserve and right ventricular output reserve. To consider the different aspects of RV reserve, parameters, or combination of parameters, should include both assessment of systolic function, as well as RV contractility, which may be approached in a stepwise estimation of RV and pulmonary arterial functions [13].

While echocardiographic assessment of right heart size is easily implemented, estimation of right heart volumes needs extensive resources such as magnetic resonance imaging machine, software and skilled radiologists. Invasive assessment of RV reserve during exercise is only feasible in expert centers with physicians who are experienced in exercise right heart catheterization. Stress-Doppler-echocardiography might therefore be more suitable for many clinics, though echocardiography during exercise also requests a thorough technique and skilled sonographers and the assessment of systolic pulmonary arterial pressure increase does only provide limited information on the RV-pulmonary arterial interaction.

Both right heart size and right ventricular reserve have shown to be of prognostic importance and may therefore be useful for risk assessment in patients with pulmonary hypertension.

## Figures and Tables

**Table 1:** Parameters covering different aspects of right ventricular reserve and function.

Parameter	Calculation	Reference Values
**Right heart catheterization**		
RV reserve [16,17]	ΔCI=CI exercise – CI rest	>20%: sensitivity 70% specificity 80% for predicting 18-month survival [16,17]
Peak CI [16,17].	Peak CI during exercise	-
Pulmonary vascular/Cardiovascular reserve/Pressure-flow relationship [13,16]	Cardiovascular reserve = mPAP/CO slope = mPAP/CO	>3 mmHg/L/min = abnormal [13,16]
End-systolic elastance [18]	$\mathrm{EEs}=\frac{\mathrm{maximal}\;\mathrm{isovolumic}\;\mathrm{pressure}-\mathrm{mPAP}}{\mathrm{SVI}}$	-
PAC [19]	$\mathrm{PAC}=\frac{\mathrm{SV}}{\mathrm{PP}}=\frac{\left(\frac{\mathrm{CO}}{\mathrm{heart}\;\mathrm{rate}}\right)}{\mathrm{sPAP}-\mathrm{dPAP}}$	Exercise PAC < 3.2 mL/mmHg: diagnosis of PAH sensitivity 90% specificity 100%
**Stress-Doppler Echocardiography**		
RV output reserve [20]	ΔsPAP =sPAP exercise−sPAP rest	<30 mmHg = worse survival
RV contractile reserve Combination of ΔTAPSE ΔRVFAC ΔS‘ [21,22]	Rest to exercise: change in TAPSE change in RV fractional area change change in Doppler-derived tricuspid lateral annular peak systolic velocity	Significantly reduced compared to controls
RV free wall strain increase [23]	Increase of strain from rest to a leg-positive pressure stress	

CI = cardiac index, CO = cardiac output, dPAP = diastolic pulmonary artery pressure, EEs = end-systolic elastance, mPAP = mean pulmonary arterial pressure, PAC = pulmonary arterial compliance, PP = pulse pressure, RV = right ventricular, sPAP = systolic pulmonary arterial pressure, SV = stroke volume, TAPSE = tricuspid annular plane systolic excursion.
